# Organization-discouraged or encouraged employee deviate behavior: a study of the typology and personal factors

**DOI:** 10.3389/fpsyg.2026.1796077

**Published:** 2026-10-14

**Authors:** Xiao-Feng Li, Chieh-Wen Sheng, Ming-Chia Chen

**Affiliations:** 1 School of General Education, Fujian Business University, Fuzhou, China; 2 Department of Information Management, Chihlee University of Technology, New Taipei, Taiwan; 3 Overseas Education College, Fujian Business University, Fuzhou, China

**Keywords:** career stage, employee deviant behavior, organizational bonding, organization-discouraged, organization-encouraged

## Abstract

**Introduction:**

It is commonly believed that employee deviant behavior should be suppressed by organizations. However, discrepancies may exist between an organization’s explicit rules and its implicit norms (Argyris and Schön, 1996). This study therefore proposes a novel typology that categorizes employee deviant behavior into actions conforming to or deviating from organizational implicit norms—termed *organization-encouraged* and *organization-discouraged* employee deviant behavior, respectively. Each category is further classified into four dimensions—personal aggression, political deviance, property deviance, and production deviance—yielding eight types in total. The study examines how personal factors, including career stage and organizational integration (affective cognition, organizational commitment, perceived obligations, and beliefs in norms), influence these eight types of behavior.

**Methods:**

A two-phase questionnaire survey was conducted. First, 100 working students described the most common deviant behaviors they had observed; these were combined with Robinson and Bennett’s (1995) 45 deviant behavior types, then coded by three scholars using content analysis (Cohen’s κ = 0.85; percentage agreement = 90%) to identify 20 typical behaviors. The survey targeted human resource managers of Taiwan’s top 1,000 enterprises, measuring career stage, organizational integration, and behavioral tendencies toward each deviant type. After two rounds of follow-up reminders, 216 valid questionnaires were collected, with no evidence of nonresponse bias (*p* > 0.05).

**Results:**

Organizations were classified as encouraging (34.72%) or discouraging (65.28%) toward deviant behavior. Members of encouraging organizations exhibited significantly higher behavioral tendencies (M = 4.24) than those in discouraging organizations (M = 3.51; F = 18.04, *p* < 0.001). Across deviant types, encouraging organizations showed significantly higher tendencies in production, political, and personal aggression deviance, but not in property deviance. Regarding career stage, tenure, subordinate count, and income predicted higher tendencies toward organization-discouraged deviance in discouraging organizations, while education and income predicted higher tendencies toward organization-encouraged deviance in encouraging organizations. For organizational integration, perceived obligations (risk and cost perceptions) significantly influenced both types of deviant behavior, whereas affective cognition, organizational commitment, and beliefs in norms did not. Hypotheses 1 and 2 were partially supported; Hypothesis 3 was not supported; Hypothesis 4 was partially supported.

**Discussion:**

This study extends Robinson and Bennett’s typology by integrating the espoused theory/theory-in-use distinction, demonstrating that implicit norms critically reshape how deviant behaviors are classified and evaluated. The partial support for hypotheses highlights the complexity of career-stage and organizational-integration effects, suggesting multiple mechanisms—socialization pressure, social learning, and perceived career risk. Practically, findings suggest that management should distinguish between organization-encouraged and organization-discouraged deviance rather than applying uniform disciplinary measures. Cross-sectional design and the Taiwanese context are limitations; future research should employ longitudinal and cross-cultural approaches.

## Background and purpose

Employee deviant behavior, also known as rule-breaking or organizational misconduct, refers to actions by organizational members that violate organizational rules and negatively impact internal management (Robinson and Bennett, 1995; Ferrell et al., 2005; Pierce and Balasubramanian, 2015; McDonnell and Nurmohame, 2021). Since these behaviors primarily affect internal management, the public outside the organization may not have sufficient information or perceive the relevant impacts. In some studies on employee deviant behavior, researchers have focused on specific deviant behaviors to explore their influencing factors, such as workplace aggression (O’Leary-Kelly et al., 1996), sales deception (MacLean, 2002), employee theft (Colquitt et al., 2006), drug abuse (Hight and Park, 2018), and attempts to delete customer and company reports (Honigsberg and Jacob, 2021). Additionally, other studies have examined multiple types of employee deviant behaviors simultaneously, as seen in the research by Stone (1978), Raelin (1984), Leatherwood and Spector (1991), Ackroyd and Thompson (1999), Vardi (2001), Sims (2002), Dore (2005), Pierce and Balasubramanian (2015), and Burbano and Ostler (2021). These studies have found that numerous factors influence employee deviant behavior, including personal, job-related, organizational, interactional factors between individuals and organizations, organizational responses to employee behavior, and even government and national cultural factors. However, these studies generally agree that, regardless of the factors involved, employee cognition is ultimately affected, leading to the initiation or suppression of various deviant behaviors.

In addition to exploring the influencing factors of employee deviant behavior, another common area of research involves the classification of such behaviors. A frequently cited study in this regard is that of Robinson and Bennett (1995), who categorized employee deviant behavior into four types: personal aggression, political deviance, production deviance, and property deviance. Subsequently, Aquino et al. (1999) confirmed and further discussed the possible types of employee deviant behavior under Robinson and Bennett’s classification. Although Robinson and Bennett's (1995) study divided employee deviant behavior into four categories, it did not address a viewpoint raised by Foerschler (1990). Foerschler pointed out a long-standing phenomenon that many have sensed: the existence of implicit norms within organizations in addition to explicit rules and policies. This aligns with the perspective of Argyris and Schon (1996), who argued that organizations have both espoused theory (explicit rules) and theory-in-use (implicit norms), and that these espoused theories and theories-in-use often contradict each other. However, past research on employee deviant behavior has generally not incorporated a comprehensive discussion of this viewpoint, resulting in a lack of clear distinction in past studies regarding whether employee deviant behavior conforms to norms.

In addition to examining influencing factors, another line of research has addressed the nature of deviant behavior by proposing categories such as unethical pro-organizational behavior (UPOB), which refers to acts intended to benefit the organization or its members while violating societal norms (Umphress and Bingham, 2011; Mishra et al., 2021); prosocial rule breaking (PSRB), which involves intentionally violating formal policies to promote organizational or stakeholder welfare (Morrison, 2006; Borry, 2023); and constructive deviance, which denotes voluntary behavior that departs from organizational norms but aims to improve organizational or member wellbeing (Galperin, 2003; Mortimer and Wang, 2021). These categories share a common insight: not all rule-violating behaviors are intended to harm the organization, and some may even be viewed favorably within certain organizational contexts.

However, despite their theoretical richness, these categories have not fully addressed the role of organizational implicit norms in shaping how deviant behaviors are classified and evaluated. UPOB and PSRB focus primarily on the intent to benefit the organization, while constructive deviance emphasizes the positive outcomes of norm violation. None of these frameworks explicitly incorporates the distinction between an organization’s espoused rules and its theory-in-use (Argyris and Schon, 1996), nor do they systematically examine how implicit norms might reclassify deviant behaviors beyond intention or outcome alone. This study addresses this theoretical blind spot by proposing a classification framework that integrates the espoused theory/theory-in-use distinction with Robinson and Bennett's (1995) typology. Specifically, we argue that ignoring the influence of implicit norms on the classification of deviant behavior leads to measurement models that conflate behaviors with fundamentally different organizational meanings.

Moreover, many studies on ethical or moral issues often employ ethical vignettes to measure the moral judgment of participants (e.g., Robertson et al., 1999; Sims, 2002; Gerwen et al., 2018). The advantage of ethical vignettes is that they provide more information, which aligns with the decision-making habits of individuals who make judgments based on multiple pieces of information. However, vignette tests also have drawbacks, including framing effects (Bateman et al., 2002) and the fact that vignettes often involve hypothetical scenarios asking participants to rate their agreement with proposed solutions. Thus, participants’ responses may only reflect a portion of their true attitudes (Paolillo and Vitell, 2002). Additionally, due to participants’ limited patience, only a few vignettes can be tested, and the vignettes used in each study are not always consistent, resulting in low external validity.

Therefore, this study first categorizes employee deviant behavior into actions that are encouraged due to conformity with organizational implicit norms and those that are discouraged due to non-conformity. It then identifies some typical or significant cases to develop a measurement questionnaire for survey research. Furthermore, since past research has generally accepted that personal factors (e.g., personal cognition) are an important influence on employee deviant behavior, this study also explores the relationship between employee deviant behavior and personal factors. The following sections first review the literature on the classification of employee deviant behavior and then discuss its influencing factors, leading to the formulation of relevant hypotheses.

## Literature review

### Typology of employee deviant behavior

Employee deviant behavior, according to Robinson and Bennett (1995), refers to the intentional violation of organizational espoused rules by employees, causing harm to the organization. This harm can be severe or minor, but given that 33–75% of employees in organizations may engage in deviant behavior (Harper, 1990), the losses incurred by organizations and industries due to employee deviant behavior are substantial (Murphy, 1993). Past research on employee deviant behavior has often focused on specific issues, such as workplace aggression (O’Leary-Kelly et al., 1996), sales deception (MacLean, 2002), sexual harassment (McCabe and Hardman, 2005), employee theft (Colquitt et al., 2006), and attempts to delete customer and company reports (Honigsberg and Jacob, 2021). Additionally, some studies have attempted to classify employee deviant behavior or integrate different types of deviant behavior for comprehensive exploration, such as those by Raelin (1984), Robinson and Bennett (1995), Aquino et al. (1999), Vardi (2001), and Burbano and Ostler (2021). Robinson and Bennett argued that previous classifications were not sufficiently comprehensive in terms of integration, and the methods used lacked inductive validity. Therefore, they employed a multidimensional scaling approach, considering the severity of behavior and its tendency to cause harm to the organization or interpersonal relationships, to reclassify employee deviant behavior into personal aggression, political deviance, property deviance, and production deviance. Following Robinson and Bennett's (1995) classification of employee deviant behavior into four types, Aquino et al. (1999) further simplified these four types into two original categories: organizational deviance, which includes production and property deviance, and interpersonal deviance, which includes personal aggression and political deviance.

These classification methods and results are essentially based on Robinson and Bennett's (1995) definition of employee deviant behavior, which states that these behaviors are voluntary actions by employees that violate organizational espoused rules and cause internal harm to the organization. However, Argyris and Schon (1996) pointed out that in addition to explicit rules (referred to as espoused theory), organizations also have implicit norms that guide daily operations (theory-in-use), and that explicit rules do not always equate to actual practices. Furthermore, Argyris and Schon argued that the implicit norms that organizational members encounter daily are the true context they face and feel, and these contexts may provide more meaningful guidance for employee behavior than the explicit rules, such as employee handbooks, which are clearly stated but not always followed. This view is also shared by Foerschler (1990) and Ashena et al. (2019). From a legal perspective, Foerschler pointed out that past court cases regarding corporate misconduct typically determined corporate liability for employee misconduct based on whether the company’s explicit policies were violated. However, Foerschler argued that even if a company’s explicit policies did not require employees to engage in illegal acts, if the implicit norms of the organization condoned or indirectly guided employees to engage in illegal or inappropriate behavior, the company should still be held responsible for such actions.

After distinguishing between an organization’s explicit rules and implicit norms, Argyris and Schon (1996) further noted that internal inquiry or questioning within an organization often helps identify and address issues. However, during this process of self-inquiry, defensive mechanisms may arise, leading the organization to lose its capacity for self-reflection. Argyris and Schon termed this phenomenon “skilled incompetence,” where organizations skillfully avoid discussing certain faults or deliberately ignore them, and these skills often exist within the organization’s daily implicit norms. Therefore, when organizational members violate explicit rules, if the organization’s implicit norms—its daily practices—deliberately overlook these deviant behaviors, it sends a signal encouraging members to continue such actions. For example, Ghosh and Shum (2019) found in their study of the hotel industry that the intentions behind employees’ rule-breaking behaviors included personal gain, improving work efficiency, helping subordinates or colleagues, and providing good customer service. Thus, as Morrison (2006) found, rule-breaking behaviors could be seen as “positive” or “constructive.”

Regarding how an organization’s daily implicit norms might encourage employee deviant behavior, McCabe and Hardman’s study on sexual harassment indicated that although sexual harassment is a form of employee deviant behavior that clearly violates organizational rules, observers of such behavior might be expected to intervene. However, McCabe and Hardman argued that due to the social influence effect within organizations—where others’ reactions to the behavior significantly affect the likelihood of intervention—once organizations fail to address members’ deviant behaviors, employees may view adherence to rules as a meaningless ritual. This can lead to employee deviant behavior being perceived as normal behavior, increasing the likelihood of continued transgressions.

Based on the literature review, this study posits that employee deviant behavior can also be classified into actions that conform to organizational implicit norms and are thus encouraged, and those that do not conform and are therefore discouraged. Table 1 presents this classification framework. First, following Robinson and Bennett's (1995) perspective, employee deviant behavior is categorized into personal aggression, political deviance, property deviance, and production deviance. Then, integrating the views of Foerschler (1990), Argyris and Schon (1996), MacLean (2002), McCabe and Hardman (2005), and Ashena et al. (2019), employee deviant behavior is further classified based on whether it conforms to organizational norms. Finally, a 4 × 2 matrix of employee deviant behavior types is formed, including organization-discouraged employee deviant behavior, which typically faces active intervention, monitoring, and handling by the organization, and organization-encouraged employee deviant behavior, which the organization may ignore, fail to address deliberately, or indirectly guide employees to engage in through certain measures.

**Table 1:** Classification framework of employee deviant behavior.

Conformity to organizational informal norms	Personal infringement behavior	Political deviant behavior	Property deviant behavior	Production deviant behavior
Non-compliant with organizational informal norms	Organizationally suppressed personal infringement behavior	Organizationally suppressed political deviant behavior	Organizationally suppressed property deviant behavior	Organizationally suppressed production deviant behavior
Compliant with organizational informal norms	Organizationally encouraged personal infringement behavior	Organizationally encouraged political deviant behavior	Organizationally encouraged property deviant behavior	Organizationally encouraged production deviant behavior

The four types of deviant behavior identified by Robinson and Bennett (1995) are reshaped differently by organizational implicit norms. In personal aggression, organization-encouraged deviance may manifest as harsh but tolerated supervisory practices that align with a ‘tough love’ culture, whereas organization-discouraged deviance involves overt hostility that triggers immediate intervention. In political deviance, encouraged behaviors might include informal coalition-building that facilitates decision-making, while discouraged behaviors involve sabotage or gossip that undermines trust. In property deviance, encouraged behaviors may include minor resource reallocation seen as pragmatic, while discouraged behaviors involve theft or destruction. In production deviance, encouraged behaviors might involve cutting corners to meet deadlines (valued as efficiency), while discouraged behaviors involve deliberate slowdowns or quality compromise. Thus, implicit norms do not uniformly reclassify all four types; rather, they interact with the specific content and consequences of each type differently.

### Influencing factors of employee deviant behavior

Research on the influencing factors of employee deviant behavior can be broadly categorized into three types: personal, organizational, and external factors. External factors include national culture and government regulations (Stone, 1978; Dore, 2005; Pierce and Balasubramanian, 2015; McDonnell and Nurmohame, 2021), while internal factors encompass supervisory oversight, organizational climate, implicit norms and historical practices, and organizational reward and punishment systems (e.g., Ashena et al., 2019). For instance, Wimbush (1999) suggested that supervisory oversight within an organization significantly impacts employee ethical behavior, particularly when employees are at a stage of moral development focused on obedience to rewards and punishments. Vardi (2001) found that when an organizational climate is characterized by warmth, support, and rewards, employee deviant behavior decreases. Foerschler (1990) emphasized the influence of implicit norms and historical actions on employee deviant behavior. Additionally, studies by Leatherwood and Spector (1991), MacLean (2002), and Lee and Ok (2014) highlighted the role of organizational reward and punishment systems in influencing employee deviant behavior. Leatherwood and Spector also mentioned that personal factors, such as employees’ considerations of ethics, costs, and risks, affect their likelihood of engaging in deviant behavior.

Employee deviant behavior is essentially the outcome of an ethical decision-making process. Cottone and Claus (2000) identified two types of factors influencing ethical decision-making: first, principle factors, where decision-makers develop certain ethical principles based on personal experience or demographic attributes to serve as criteria for ethical dilemmas; second, social interaction factors, where decision-makers are influenced by situational factors at the time of decision-making, leading to different cognitions and considerations, such as evaluating personal or group gains and losses, the demands of other stakeholders, and current and future national laws or economic conditions. Raelin (1984) also noted in his integrated model of the influencing factors of employee deviant behavior that whether organizational or job-related factors, they ultimately relate to employees’ cognitive states, expectations, or personal career stages. Therefore, this study considers personal factors to be crucial in influencing employee deviant behavior.

Previous studies have suggested that younger individuals with lower income and less industry experience, who are not yet accustomed to the ‘reality of business,’ tend to have higher ethical decision-making (Singhapakdi et al., 1996; Roozen et al., 2001). However, contemporary research on organizational socialization suggests a more nuanced mechanism. Newcomers, lacking established social networks and organizational knowledge, may face stronger pressure to conform to both formal and informal expectations, potentially increasing their susceptibility to engage in deviant behaviors—particularly those that are organizationally encouraged—as a strategy for gaining acceptance (Saks and Gruman, 2012; Bauer et al., 2007). Conversely, employees at later career stages, with greater tenure and organizational embeddedness, may feel more confident in navigating organizational norms and thus selectively engage in deviant behaviors they perceive as aligned with implicit expectations. Furthermore, social learning theory (Bandura, 1977) suggests that employees learn acceptable behaviors by observing colleagues and supervisors; the longer they are in the organization, the more exposed they become to implicit norms that may encourage certain forms of deviance. Based on these competing theoretical arguments, this study proposes that career stage may influence deviant behavior tendencies through multiple mechanisms (Li, 2024), including socialization pressure, social learning, and perceived career risk. Hypotheses 1 and 2 thus examine whether career stage is associated with organization-discouraged and organization-encouraged deviant behavior, without presuming a simple linear direction.

*Hypothesis 1*: The earlier an employee is in their career stage, the lower their tendency to engage in organization-discouraged deviant behavior.

*Hypothesis 2*: The earlier an employee is in their career stage, the lower their tendency to engage in organization-encouraged deviant behavior.

In addition to personal background data, numerous studies have indicated that personal cognition is related to employee deviant behavior. Hirschi's (1969) social bonding theory, originating from criminology, posits that individuals with stronger social bonds are less likely to engage in deviant behavior. However, to apply this framework to organizational contexts, it is necessary to bridge it with modern organizational theories. Organizational identification (OID), defined as the degree to which an employee defines themselves in terms of their membership in the organization (Mael and Ashforth, 1992), shares conceptual parallels with Hirschi’s attachment dimension. Similarly, affective commitment (Allen and Meyer, 1990)—the emotional attachment to the organization—parallels the commitment dimension of social bonding. Social exchange theory (SET; Blau, 1964; Cropanzano and Mitchell, 2005) further explains that employees reciprocate organizational treatment; when they perceive the organization as supportive, they feel obligated to contribute positively, potentially reducing organization-discouraged deviance but possibly increasing organization-encouraged deviance as a form of reciprocation. Building on these theoretical bridges, this study incorporates measures of employees’ organizational integration, including organizational affect (emotional perception of the organization), organizational commitment, perceived obligations (awareness of consequences and costs of deviant behavior), and beliefs in organizational norms. Following Sims' (2002) suggestions, perceived obligations are measured in terms of risk and cost perceptions rather than intention to leave. Additionally, beliefs in organizational norms capture the degree to which employees accept and internalize the organization’s implicit standards. Hypothesis 3 proposes that stronger organizational integration reduces tendencies toward organization-discouraged deviant behavior, as employees with higher identification, commitment, and norm beliefs are more likely to align with organizational expectations. Hypothesis 4 proposes that stronger organizational integration increases tendencies toward organization-encouraged deviant behavior, as employees may engage in such behaviors as a form of reciprocation or to maintain alignment with implicit norms.

From these studies on employee cognition and deviant behavior, it is evident that many researchers focus on whether employees’ perceptions of the organization lead to deviant behavior. For instance, expectations of the organization (Raelin, 1984), judgments of the organization’s detection capabilities (Leatherwood and Spector, 1991), and perceptions of organizational stress and opportunities (MacLean, 2002). Although employees’ affective cognition and commitment to the organization significantly influence their deviant behavior, their perceived obligations regarding deviant behavior are not significantly related. Sims (2002) suggested that this might be because, in his study, he used employees’ intention to leave as a measure of their perceived obligations regarding deviant behavior, assuming that higher intention to leave corresponds to lower perceived obligations. However, Sims later found that intention to leave did not align with Hirschi's (1969) definition of perceived obligations. Instead, the considerations of costs and risks mentioned by Leatherwood and Spector (1991) were more akin to employees’ perceived obligations regarding deviant behavior, i.e., employees’ perceptions of the consequences and costs of deviant behavior. Additionally, Sims noted that his study primarily used vignette tests to examine specific employee deviant behaviors, thus limiting the generalizability of his findings. Beyond the limitations mentioned by Sims (2002), studies by Hirschi (1969); Hollinger (1986), and Sims did not specifically distinguish between implicit cultural norms and explicit legal regulations. However, later studies, including those by Foerschler (1990); Argyris and Schon (1996); MacLean (2002); McCabe and Hardman (2005); Dore (2005), and McDonnell and Nurmohame (2021), have pointed out that explicit laws and regulations are not equivalent to implicit cultural norms, and these implicit cultural norms may have a greater impact on individuals. That is, if an individual has a high degree of social integration, they may have a higher affective cognition of society, greater commitment, higher perceived obligations, or stronger beliefs in norms. Similarly, inconsistencies between implicit norms and explicit regulations can also occur within organizations (Argyris and Schon, 1996). This may lead to a situation where stronger organizational integration increases the likelihood of engaging in deviant behaviors that are condoned or encouraged by organizational norms. Therefore, this study examines the impact of employees’ organizational integration on both organization-discouraged and organization-encouraged deviant behaviors. In addition to redesigning the items measuring employees’ perceived obligations regarding deviant behavior based on Sims' (2002) suggestions, this study also incorporates measures of employees’ beliefs in norms, which were not included in Sims’ research, and uses a questionnaire survey to test the third and fourth hypotheses:

*Hypothesis 3*: The stronger an employee’s organizational integration, the lower their tendency to engage in organization-discouraged deviant behavior.

*Hypothesis 4*: The stronger an employee’s organizational integration, the higher their tendency to engage in organization-encouraged deviant behavior.

## Research methodology

### Research framework

Following the literature review, this study proposes four hypotheses, with the sub-hypotheses detailed in Table 2. Figure 1 illustrates the relationships among the four hypotheses, i.e., the research framework. This study intends to validate these hypotheses using a questionnaire survey. To ensure respondents understand the employee deviant behaviors mentioned in the questionnaire, the behaviors should ideally be ones that respondents have observed or heard of within their organizations, i.e., typical or commonly occurring employee deviant behaviors.

**Table 2:** Organizational attitudes towards employee misconduct.

Type	Count	Percentage	Valid percentage	Cumulative percentage
Encourage	75	34.72	34.72	34.72
Discourage	141	65.28	65.28	100.00
Total	216	100.00	100.00	

**Figure 1:**
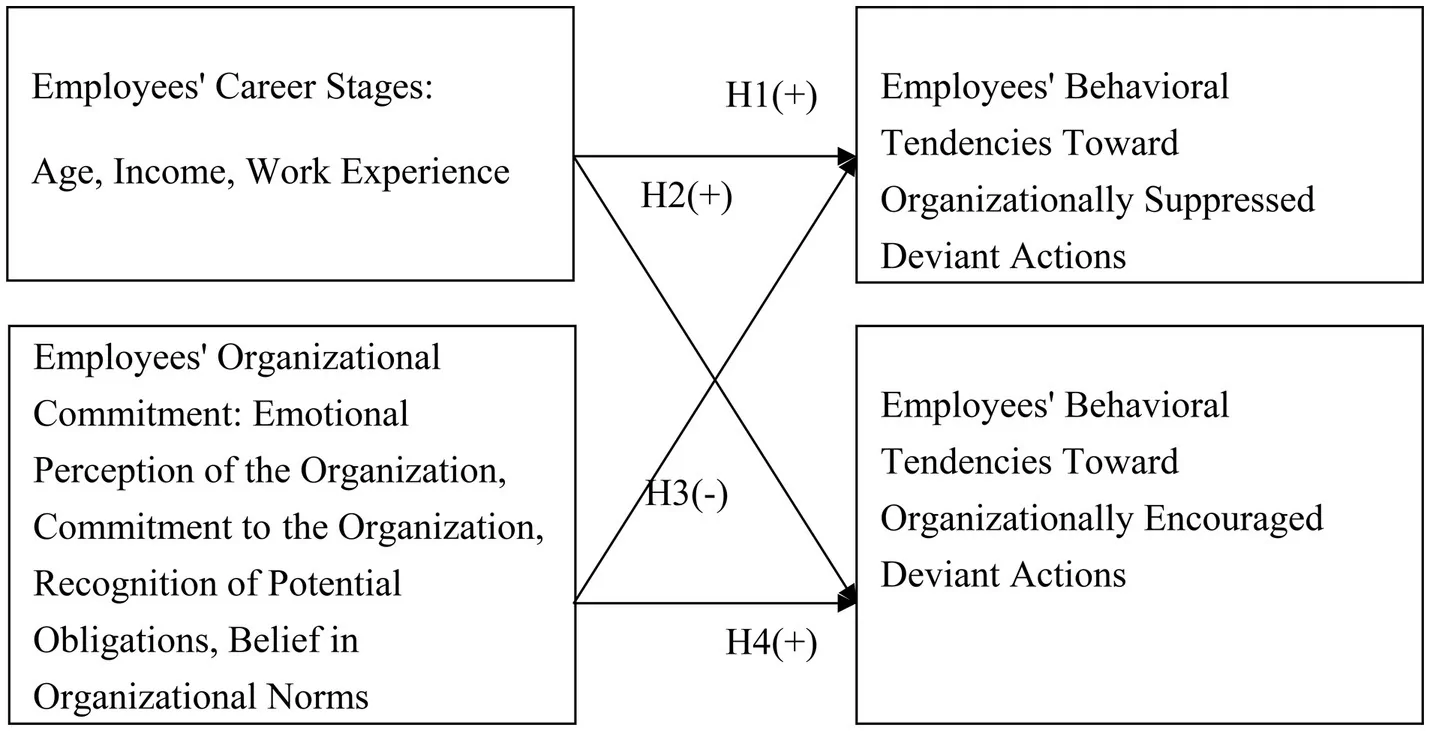
Research framework.

Therefore, this study first identifies typical employee deviant behaviors to design questionnaire items, measures the variables, and analyzes the relationships to test the hypotheses. The methodology is described below.

### Identification of typical employee deviant behaviors

In Robinson and Bennett's (1995) study, 45 types of employee deviant behaviors were listed, including falsifying overtime hours, verbally abusing colleagues, intentionally making mistakes, tardiness, and damaging equipment. They classified these behaviors into four categories. Given the reasonableness and rigor of their classification, this study adopts their categorization, dividing employee deviant behavior into personal aggression, political deviance, property deviance, and production deviance. However, this study also recognizes Dore's (2005) perspective that different national cultures affect individuals’ cognitions. Thus, this study incorporates a Taiwanese viewpoint by using purposive sampling to ask 100 local working students who have studied business ethics to describe the most common employee deviant behaviors they have observed in organizations. These behaviors are then combined with the 45 types mentioned by Robinson and Bennett and listed accordingly.

Subsequently, this study uses content analysis to process the collected employee deviant behaviors. Three scholars with relevant work experience in fields related to business ethics or employee deviant behavior are invited to code these behaviors based on Robinson and Bennett's (1995) perspectives, categorizing each deviant behavior into personal aggression, political deviance, property deviance, or production deviance. Prior to coding, detailed category definitions are provided to the coders. After coding, the inter-coder reliability is calculated to be 0.90, and the reliability is 0.85, indicating acceptable coding.

Prior to coding, detailed category definitions were provided to the coders, including examples of each type of deviant behavior. The inter-coder reliability was calculated using Cohen’s kappa (*κ* = 0.85), and the percentage agreement was 90%, both indicating acceptable coding reliability (Cohen, 1960; Lombard et al., 2002). Discrepancies between coders were resolved through discussion with the lead researcher.

Finally, this study calculates the frequency of each deviant behavior mentioned by the 100 working students. Within each category (personal aggression, political deviance, property deviance, and production deviance), the five most frequently mentioned behaviors are selected as typical or most commonly observed employee deviant behaviors. The determination of whether these behaviors are organization-discouraged or organization-encouraged will be addressed during questionnaire design.

### Questionnaire design and survey analysis

Regarding questionnaire design, this study targets the 20 typical employee deviant behaviors identified across four categories. For each behavior, four questions are designed to assess respondents’ tendencies towards organization-discouraged or organization-encouraged deviant behavior. The first and second questions aim to measure respondents’ tendencies towards organization-discouraged or organization-encouraged deviant behavior. The first question assesses the degree to which the organization encourages the deviant behavior; stricter organizational responses indicate stronger discouragement or suppression of the behavior. For two typical behaviors within each category, including those more discouraged and more encouraged by the organization, respondents’ answers to the second question are recorded to gauge their likelihood of engaging in the behavior, i.e., their behavioral tendency. Thus, for organization-discouraged deviant behaviors, if respondents indicate that the behavior is acceptable under reasonable circumstances, it suggests a higher tendency towards the behavior. Conversely, strong disapproval indicates a lower tendency. To reduce social desirability bias, the study employs anonymous questionnaires and, following Vardi's (2001) suggestion, asks respondents to rate their agreement with observed behaviors rather than their own actions, indirectly assessing their tendencies towards organization-discouraged or organization-encouraged deviant behavior. The third and fourth questions for each deviant behavior focus on measuring employees’ perceived obligations regarding the behavior. Drawing on the concepts of cost and risk proposed by Leatherwood and Spector (1991), and recognizing that different behaviors entail varying costs and risks, the questionnaire asks respondents to assess the perceived risks (third question) and costs (fourth question) associated with organization-discouraged or organization-encouraged deviant behavior. These assessments are then statistically analyzed alongside respondents’ behavioral tendencies (second question).

In addition to validating Hypotheses 3 and 4, for Hypotheses 1 and 2, the study measures employees’ career stages using age, income, and length of industry work experience, following the perspectives of Raelin (1984) and Roozen et al. (2001), and examines their relationship with employee deviant behavior (whether conforming to or deviating from norms). Data on age, income, and industry work experience are collected as part of respondents’ personal background information, and the questionnaire also requests some basic organizational data.

After questionnaire completion and necessary semantic feedback and pre-testing, the survey targets human resource managers of the top 1,000 enterprises in Taiwan. Human resource departments are responsible for establishing organizational systems and handling employee behavior rewards and punishments, thus having a deep understanding of various employee deviant behaviors and organizational norms. After mailing the questionnaires, follow-ups are conducted every 2 weeks, with a total of two reminders. Upon questionnaire collection, in addition to organizing respondents’ background data, an analysis of nonresponse bias is performed. According to Armstrong and Overton's (1977) assumption that late respondents are similar to non-respondents, the first and last quartiles of respondents are compared in terms of demographic data and other variables to check for significant differences in background or variable distributions, confirming the absence of nonresponse bias. A total of 216 valid questionnaires were collected, with 144 responses received before follow-up and 72 after. The bias test (*p* > 0.05) showed no significant differences.

## Research results

### Distinction between organization-discouraged and organization-encouraged employee deviant behavior

Based on the collected data, organizations’ attitudes towards employee misconduct are divided into two groups: encouraging and discouraging. Among them, 75 organizations (34.72%) are classified as encouraging, while 141 organizations (65.28%) are classified as discouraging (Table 2). Comparative analysis (Table 3) reveals that members of encouraging organizations exhibit significantly higher tendencies towards misconduct compared to those in discouraging organizations, indicating a clear distinction between the two groups. Further analysis across different types of deviant behavior shows that, except for property deviance, organizations tend to encourage rather than discourage production, political, and personal aggression deviance (Table 4).

**Table 3:** Employee tendencies towards organization-encouraged or discouraged deviant behavior.

Dependent variable	Organizational type	Mean	Std. deviation	Std. error mean	*F*-value	*p*-value
Behavioral intention	Encourage	4.24	0.31	0.04	18.04	0.00^*^
	Discourage	3.51	0.56	0.05		

^*^p < 0.05.

**Table 4:** Organization-discouraged or encouraged employee deviant behavior.

Deviant type	Organizational type	*N*	Mean	Std. deviation	Std. error	*F*-test	Sig.
Production deviance	Encourage	75.00	3.63	0.44	0.05	5.20	0.02
	Discourage	141.00	2.87	0.60	0.05		
Property deviance	Encourage	75.00	4.13	0.39	0.04	1.14	0.29
	Discourage	141.00	3.48	0.45	0.04		
Political deviance	Encourage	75.00	4.21	0.37	0.04	16.21	0.00
	Discourage	141.00	3.50	0.59	0.05		
Personal aggression	Encourage	75.00	4.84	0.23	0.03	22.73	0.00
	Discourage	141.00	4.35	0.53	0.04		

### Examination of career stage

The study separately tested the impact of career stage on employee misconduct tendencies within discouraging and encouraging organizations (Tables 5, 6). In discouraging organizations, higher tenure, subordinate count, and monthly income were associated with higher tendencies towards misconduct. In encouraging organizations, higher education level and monthly income were linked to higher misconduct tendencies. Thus, Hypotheses 1 and 2 are partially supported.

**Table 5:** Regression analysis of career stage on organization-discouraged deviant behavior.

Regression	Sum of squares	df	Mean square	*F*	Sig.
	5.84	8.00	0.73	2.53	0.01

Predictor	Unstandardized coefficients		Standardized coefficients	*t*	Sig.
	Estimate	Std. error	Beta		
Tenure	0.02	0.01	0.34	2.17	0.03
Subordinates	0.02	0.01	0.36	3.61	0.00
Monthly income	0.13	0.06	0.23	2.04	0.04

**Table 6:** Regression analysis of career stage on organization-encouraged deviant behavior.

Regression	Sum of squares	df	Mean square	*F*	Sig.
	1.55	8.00	0.19	2.29	0.03

Predictor	Unstandardized coefficients		Standardized coefficients	*t*	Sig.
	Estimate	Std. error	Beta		
Education	0.14	0.06	0.32	2.42	0.02
Monthly income	0.14	0.05	0.51	−2.70	0.01

### Examination of organizational integration

Regarding the impact of organizational integration variables—such as beliefs in norms, affective cognition, organizational commitment, and perceived obligations—on employee misconduct tendencies, the results show that regardless of whether the organization is discouraging or encouraging, perceived obligations (risk and cost perceptions) have significant effects, while beliefs in norms, affective cognition, and organizational commitment do not (Tables 7, 8). Thus, Hypothesis 3 is not supported, while Hypothesis 4 is partially supported.

**Table 7:** Regression analysis of organizational integration on organization-discouraged deviant behavior.

Regression	Sum of squares	df	Mean square	*F*	Sig.
	16.28	5.00	3.26	15.93	0.00

Predictor	Unstandardized coefficients		Standardized coefficients	*t*	Sig.
	Estimate	Beta	Beta		
Risk perception	0.47	0.07	0.50	6.59	0.00
Cost perception	0.29	0.12	0.19	2.39	0.02

**Table 8:** Regression analysis of organizational integration on organization-encouraged deviant behavior.

Regression	Sum of squares	df	Mean square	*F*	Sig.
	2.55	5.00	0.51	7.66	0.00

Predictor	Unstandardized coefficients		Standardized coefficients	*t*	Sig.
	Estimate	Beta	Beta		
Risk perception	0.28	0.06	0.44	4.31	0.00
Cost perception	0.34	0.11	0.30	2.94	0.00

## Research implications

The findings of this study carry several important theoretical, practical, and policy implications. Theoretically, this study extends Robinson and Bennett's (1995) typology by integrating the espoused theory/theory-in-use distinction (Argyris and Schon, 1996), demonstrating that organizational implicit norms play a critical role in reshaping how deviant behaviors are classified and evaluated. This framework complements existing categories such as UPOB (Umphress and Bingham, 2011; Mishra et al., 2021; Luan et al., 2023), PSRB (Morrison, 2006; Borry, 2023), and constructive deviance (Galperin, 2003; Mortimer and Wang, 2021), while offering a distinct lens that focuses on the alignment between behavior and implicit organizational expectations rather than intent or outcome alone.

Practically, our findings suggest that organizational leaders and HR managers should recognize the dual nature of deviant behavior. Not all rule-violating behaviors are equally harmful, and some may reflect adaptive responses to implicit organizational expectations. Management policies should therefore distinguish between organization-encouraged and organization-discouraged deviant behaviors, rather than applying uniform disciplinary measures. From a policy perspective, organizations should strive to align their formal policies (espoused theory) with their informal practices (theory-in-use) to reduce ambiguity in behavioral expectations and minimize the occurrence of genuinely harmful deviant behaviors.

Several limitations of this study should be acknowledged. First, the cross-sectional design limits causal inference. Future research should employ longitudinal designs to examine how implicit norms evolve over time and how they influence deviant behavior trajectories. Second, the study is situated in the Taiwanese organizational context; cross-cultural comparisons would enhance the generalizability of the findings. Third, the classification of deviant behaviors into encouraged and discouraged categories was based on content analysis of questionnaire responses; future research should employ multi-source validation, including interviews with employees across different industries, to strengthen the robustness of the typology. Fourth, the partial support for our hypotheses regarding career stage suggests that the relationship between career stage and deviant behavior is more complex than initially theorized, involving multiple mechanisms such as socialization pressure, social learning, and perceived career risk. Future research should explore these mechanisms through mediation and moderation analyses.

Future research could also benefit from incorporating qualitative methods, such as in-depth interviews and ethnographic observations, to uncover the specific processes through which implicit norms are communicated, internalized, and enacted in different organizational settings. Additionally, examining the role of leadership styles, organizational culture, and industry characteristics in shaping implicit norms would provide a more comprehensive understanding of the contextual factors that influence employee deviant behavior.

## Research conclusions

The findings indicate that employees’ tendencies towards organization-encouraged deviant behavior and their perceived costs are significantly higher than those towards organization-discouraged deviant behavior. Regarding career stage, higher tenure, subordinate count, and monthly income are associated with higher tendencies towards organization-discouraged deviant behavior, while higher education level and monthly income are linked to higher tendencies towards organization-encouraged deviant behavior.

Furthermore, organizational integration, particularly perceived obligations (risk and cost perceptions), positively influences tendencies towards both organization-discouraged and organization-encouraged deviant behavior. This contradicts Hypothesis 3, which posited that stronger organizational integration would reduce tendencies towards organization-discouraged deviant behavior. Perceived obligations refer to employees’ awareness of the consequences and costs of deviant behavior. If employees perceive higher potential costs, they are less likely to engage in deviant behavior. Conversely, if employees perceive lower potential costs and a lower likelihood of detection, their tendency towards deviant behavior increases. Since the most common deviant behaviors identified in this study are relatively minor, clearer perceptions of obligations lead to higher tendencies towards such behaviors.

The findings of this study underscore the importance of considering organizational implicit norms when examining employee deviant behavior. While traditional frameworks have focused on formal rules as the primary basis for classifying deviance, our results suggest that implicit norms play a significant role in shaping how organizations categorize and respond to deviant behaviors. The partial support for our hypotheses highlights the complexity of the relationships between career stage, organizational integration, and deviant behavior, suggesting that future research should explore these relationships through multiple theoretical lenses, including socialization, social learning, and social exchange perspectives.

## Data Availability

The original contributions presented in the study are included in the article/supplementary material, further inquiries can be directed to the corresponding author.
